# Nanoparticle T-cell engagers as a modular platform for cancer immunotherapy

**DOI:** 10.1038/s41375-021-01127-2

**Published:** 2021-01-21

**Authors:** Kinan Alhallak, Jennifer Sun, Katherine Wasden, Nicole Guenthner, Julie O’Neal, Barbara Muz, Justin King, Daniel Kohnen, Ravi Vij, Samuel Achilefu, John F. DiPersio, Abdel Kareem Azab

**Affiliations:** 1 Department of Radiation Oncology, Washington University School of Medicine, St. Louis, MO USA; 2 Department of Biomedical Engineering, Washington University, St. Louis, MO USA; 3 Department of Medicine, Washington University School of Medicine, St. Louis, MO USA; 4 Department of Radiology, Washington University School of Medicine, St. Louis, MO USA

**Keywords:** T cells, Myeloma, Lymphoma, Biotechnology

## Abstract

T-cell-based immunotherapy, such as CAR-T cells and bispecific T-cell engagers (BiTEs), has shown promising clinical outcomes in many cancers; however, these therapies have significant limitations, such as poor pharmacokinetics and the ability to target only one antigen on the cancer cells. In multiclonal diseases, these therapies confer the development of antigen-less clones, causing tumor escape and relapse. In this study, we developed nanoparticle-based bispecific T-cell engagers (nanoBiTEs), which are liposomes decorated with anti-CD3 monoclonal antibodies (mAbs) targeting T cells, and mAbs targeting the cancer antigen. We also developed a nanoparticle that targets multiple cancer antigens by conjugating multiple mAbs against multiple cancer antigens for T-cell engagement (nanoMuTEs). NanoBiTEs and nanoMuTEs have a long half-life of about 60 h, which enables once-a-week administration instead of continuous infusion, while maintaining efficacy in vitro and in vivo. NanoMuTEs targeting multiple cancer antigens showed greater efficacy in myeloma cells in vitro and in vivo, compared to nanoBiTEs targeting only one cancer antigen. Unlike nanoBiTEs, treatment with nanoMuTEs did not cause downregulation (or loss) of a single antigen, and prevented the development of antigen-less tumor escape. Our nanoparticle-based immuno-engaging technology provides a solution for the major limitations of current immunotherapy technologies.

## Introduction

Cancer immunotherapy improves the ability of the immune system to recognize and combat cancer cells, which enables long-term remission in cancer patients and is also in the forefronts of cancer therapy [1, 2]. T-cell-based immunotherapies include chimeric antigen receptor T (CAR-T) cells and bispecific T-cell Engagers (BiTEs). CAR-T cells are autologous T cells obtained from individual patients and are genetically engineered to express an antibody single-chain variable fragment (scFv) to recognize and kill cancer [3]. BiTEs are tandem scFv fragments connected by flexible linkers with one scFv targeting a T-cell specific molecule such as CD3, while the other targets a tumor-associated antigen, which allows the BiTEs to redirect the T cell to the cancer cell, leading to T-cell-redirected activation and tumor killing [4–7].

T-cell-based immunotherapy has shown promising clinical outcomes in many cancers including multiple myeloma (MM) [4, 5] and Waldenstrom macroglobulinemia (WM) [8]; however, these have significant limitations. CAR-T cells must be extracted from the patient, activated, expanded, genetically engineered, and purified ex vivo for reinjection into the patient [9, 10]. This process imposes technical challenges and significant expense. BiTEs, on the other hand, have the advantage of being off the shelf for immediate use in patients [11]; however, they have a poor pharmacokinetic profile, with a half-life of around 2 h [12], imposing compromised patient quality of life, and increased risk of infections related deaths [13–16]. Therefore, there is an urgent need to develop new forms of T-cell immunotherapies that overcome these limitations.

## Results and discussion

**Fig. 1:**
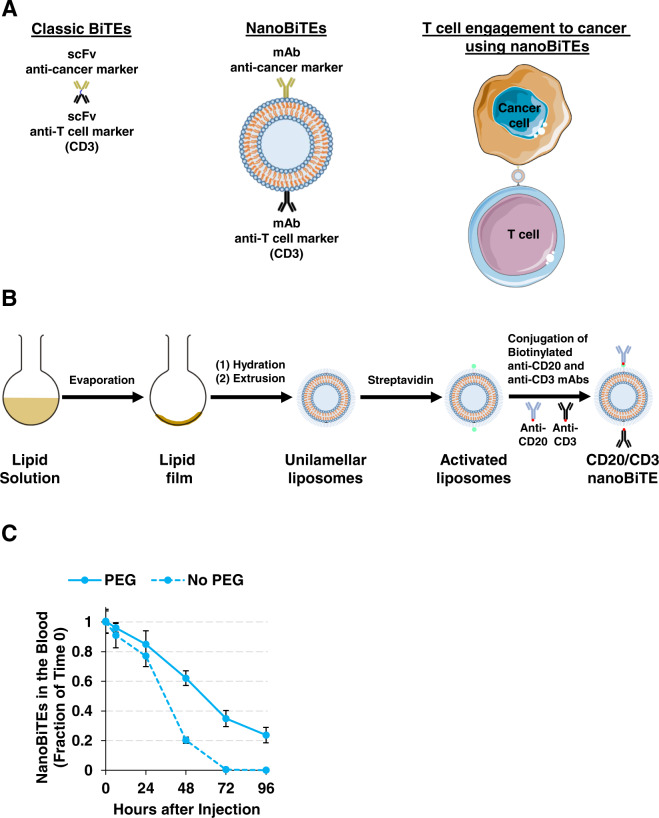
Development of nanoparticle bispecific T-cell engagers (nanoBiTEs). **A** Schematic of classic BiTEs, nanoparticle T-cell engagers (nanoBiTEs), and the utilization of nanoBiTE to engage T cells to cancer cells. **B** A scheme of the production of the nanoBiTE using thin-film evaporation method, followed by conjugation of mAbs of choice such as anti-CD20 and anti-CD3. **C** The pharmacokinetic profile of nanoBiTEs with or without PEGgylation in vivo (*n* = 3; means ± SD).

We have developed nanoparticle-based BiTE (nanoBiTEs), which are liposomes decorated with anti-CD3 monoclonal antibodies (mAbs) targeting T cells, and mAbs targeting the cancer antigen (Fig. 1A). We hypothesized that the liposomal nature of nanoBiTEs will have a prolonged half-life. We developed liposomes with or without stealth PEGylation conjugated to these anti-CD3 and anti-CD20 mAbs (CD20/CD3 nanoBiTEs) (Fig. 1B). We chose to target CD20 for targeting WM cells, since CD20 has been routinely and successfully used as a therapeutic target for WM [17, 18]. Non-PEGylated nanoBiTEs improved the half-life to about 36 h, while the PEGylated nanoBiTEs had even a longer half-life of about 60 h (Fig. 1C). Therefore, we adopted the PEGylated nanoBiTEs formulation for all upcoming experiments. The longer half-life enabled administration of the nanoBiTEs once a week as an intravenous (IV) bolus injection for in vivo experiments. Clinically, the improved pharmacokinetic profile will be translated into a more convenient dosing regimen and therefore a dramatic improvement in the patient’s quality of life, and decrease risk of infections related to continuous infusion. Other solutions that have been established to circumvent the low pharmacokinetic profile include supplementing BiTEs with an Fc receptor or an antihuman serum albumin binding construct [19, 20]; both methods prevent the rapid elimination and degradation of BiTEs by the neonatal Fc receptor [21, 22]. There are currently multiple ongoing clinical trials testing these newly designed BiTEs for efficacy and toxicity [23–27].

**Fig. 2:**
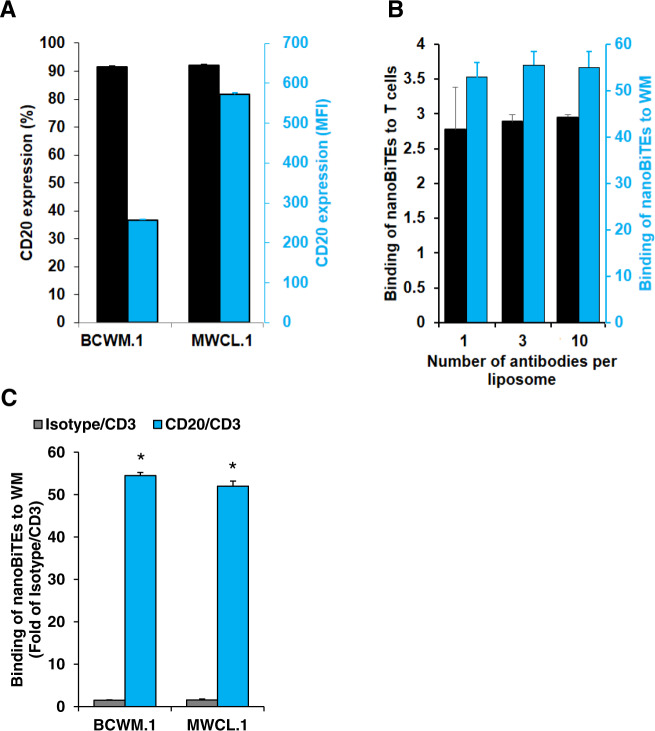
Development of nanoBiTEs for Waldenstrom macroglobulinemia (WM). **A** RMFI and percent of CD20 protein expression on the surface of WM cells (*n* = 3; means ± SD). **B** The effect of the number of anti-CD20 mAbs conjugated to the liposome on the binding of the nanoBiTEs to BCWM.1 cells, and the effect of the number of anti-CD3 mAbs conjugated to the liposome on the binding of the nanoBiTEs to T cells (*n* = 3; means ± SD). **C** Binding of isotype/CD3 and CD20/CD3 nanoBiTEs to WM cells (*n* = 3; means ± SD). Two-sided Student’s *t* test was used; statistical significance (*p*  < 0.05) between CD20/CD3 and isotype/CD3 was indicated by placing an asterisk.

First, we validated the use of CD20 as a target for the treatment of WM. We measured the percent of WM cells that express CD20. For both WM cell lines, CD20 is highly expressed and on ~90% of cells (Fig. 2A). We then investigated the effect of the number of antibodies conjugated to the liposome. Increasing the number of antibodies conjugated to the liposomes did not increase the binding of the nanoBiTEs to WM or T cells, which is shown in Fig. 2B. Therefore, for all the upcoming experiments, we developed nanoBiTEs with one CD3 and one CD20 mAb per liposome. We then tested the binding of the CD20/CD3 nanoBiTEs to WM cells, compared to isotype and CD3 conjugated nanoBiTEs (isotype/CD3). The CD20/CD3 nanoBiTEs bound to the WM cells about 50-fold greater than isotype/CD3 nanoBiTEs (Fig. 2C).

**Fig. 3:**
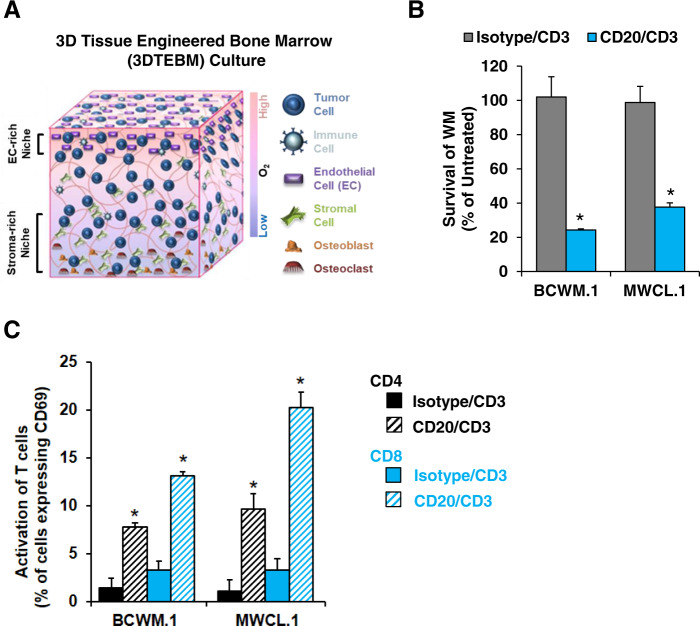
WM killing and T-cell activation with nanoBiTEs in vitro. **A** A scheme of 3DTEBM cultures used to determine the effect of nanoBiTEs on T-cell activation and cancer cell killing in vitro. **B** The effect of isotype/CD3 and CD20/CD3 nanoBiTEs on the killing of WM cells with T cells (*n* = 4; means ± SD). **C** The effect of isotype/CD3 and CD20/CD3 nanoBiTEs on the expression of CD69 on CD4+ and CD8+ T cells as a marker of T-cell activation (*n* = 3; means ± SD). Two-sided Student’s *t* test was used; statistical significance (*p* < 0.05) between CD20/CD3 and isotype/CD3 was indicated by placing an asterisk.

To demonstrate the therapeutic efficacy of the nanoBiTEs, we used our 3D tissue-engineered bone marrow (3DTEBM) model [28] (Fig. 3A), in which we used primary BM aspirates from patients to develop a 3D culture of the malignant BM niche. The model is developed using all the cells in the tumor microenvironment, not only tumor cells, but also other accessory cells including T cells. We used the BM supernatant from patients to create the 3D matrix by cross-linking fibrinogen naturally found in the marrow; the cellular fraction is also reintroduced into the scaffold. The 3DTEBM recapitulates cellular structures and oxygen gradients of the BM niche, and allows proliferation of primary cells from various hematologic malignancies (such as WM and MM). It can be also used with cell lines in combination with the tumor microenvironment (without cancer cells) isolated from patients. We suggest this model as an optimal model for testing the effect of T-cell-based immunotherapies in vitro.

We tested the effect of CD20/CD3 nanoBiTEs on the survival of WM cells in the 3DTEBM. CD20/CD3 nanoBiTEs induced 60–70% killing of WM cells, while the isotype/CD3 nanoBiTEs did not induce any killing whatsoever (Fig. 3B). We ensured that the MM cell lysis seen with nanoBiTEs was T cell mediated by incubating WM cells and nanoBiTEs or isotype/CD3 without T cells and observed no killing of WM cells as seen in Supplementary Fig. 1. In addition, we tested the activation of T cells by nanoBiTEs in the 3DTEBM. CD69 expression, as a marker of T-cell activation, in CD4 and CD8 T cells (Fig. 3C) was higher after treatment with CD20/CD3 nanoBiTE compared to isotype/CD3. Moreover, the CD8 T cells showed higher activation compared to CD4 T cells. Secretion of cytokines is a hallmark of T-cell activation. Supplementary Fig. 2 shows the cytokine secretion of T cells following their activation with CD20/CD3 and isotype/CD3 nanoBiTEs in the 3DTEBM. The presence of IL-2, IL-6, IL-10, TNF-α, and IFN-γ is significantly greater when treated with CD20/CD3 compared to isotype/CD3. These results indicate that the CD20/CD3 nanoBiTEs are specific to WM cells and that the effect is only mediated via T-cell engagement.

**Fig. 4:**
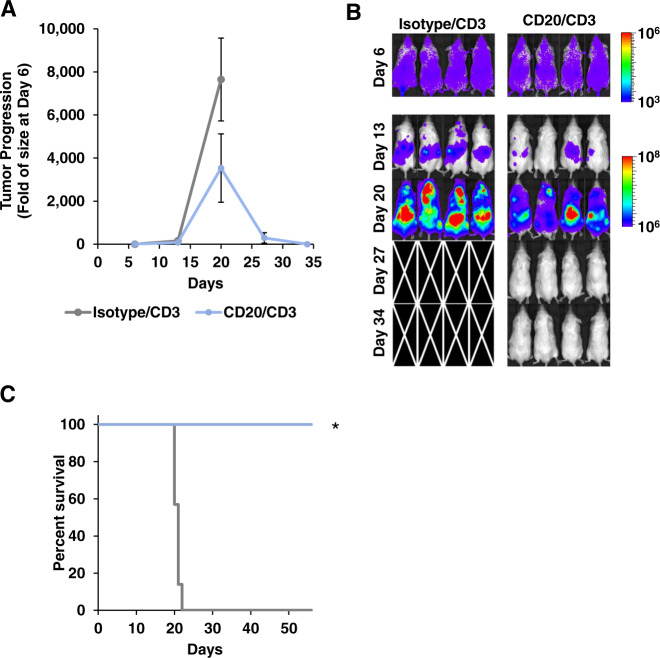
WM killing and T-cell activation with nanoBiTEs in vitro. **A** Quantitative and **B** qualitative analysis of the effect of isotype/CD3 and CD20/CD3 nanoBiTEs on the progression of WM tumors in vivo (*n* = 7; means ± SEM). **C** The effect of isotype/CD3 and CD20/CD3 nanoBiTEs on the survival of WM-bearing mice (*n* = 3). Log-rank test was used to compare the Kaplan–Meier curves; statistical significance (*p*  < 0.05) between CD20/CD3 and isotype/CD3 was indicated by placing an asterisk.

To demonstrate the therapeutic efficacy in vivo, we chose an aggressive xenograft WM model by injecting BCWM.1 cells IV (with humanized T cells), which kills the mice in less than 3 weeks following injection, if not treated, this represents the clinically aggressive/relapsed form of the disease. Mice treated with isotype/CD3 nanoBiTEs showed fast tumor progression and death of the entire cohort within 21 days. In contrast, mice treated with WM-targeting CD20/CD3 nanoBiTE showed slower tumor progression at days 14 and 21, a significant reduction at day 28 (compared to day 21), and complete eradication of the tumor by day 35 (Fig. 4A, B). The entire cohort survived with no signs of disease for as long as 2 months, which is when the experiment was stopped (Fig. 4C). These results demonstrate that the CD20/CD3 nanoBiTE immunotherapy has an outstanding potential to treat/cure even the most aggressive forms of WM.

The second major limitation of BiTEs (and CAR-T cells) is that they are designed to target only one antigen on cancer cells. Preclinical studies have demonstrated that targeting multiple antigens by CAR-T cells or BiTEs are still technically challenging [29–33]. Especially in a multiclonal disease like MM [34–37], these therapies confer the development antigen-less clones, causing tumor escape and relapse of the disease [4, 5].

**Fig. 5:**
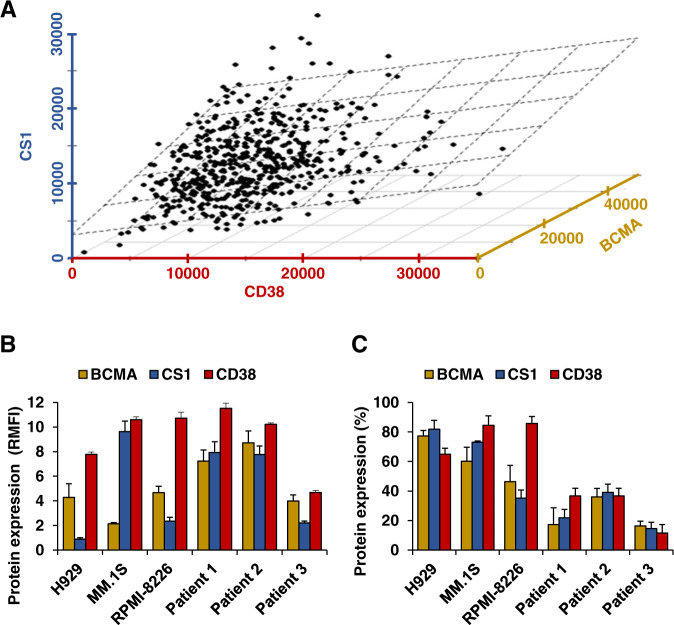
BCMA, CS1, and CD38 expressions on primary MM cells and MM cell lines. **A** mRNA gene expressions of BCMA, CS1, and CD38 in a cohort of 600 MM patients (*n* = 600). **B** RMFI and **C** percent protein expression of BCMA, CS1, and CD38 on MM cell lines and three patient primary cells (*n* = 3; means ± SD).

Several antigens were used previously as targets for T-cell-based immunotherapy in MM, including B-cell maturation antigen (BCMA), CD38, and SLAMF7 (CS1) [38–42]. Gene expression analysis of these antigens in MM patients showed that the expression of each marker was highly variable, emphasizing the heterogeneity of the expression of these genes in MM patients (Fig. 5A). We also tested the surface protein expression of these antigens on MM cells, which further showed and emphasized the variability and presence of expression (Fig. 5B, C). Such heterogeneous expression presents a challenge for the efficacy of any immunotherapy that targets any of these antigens as a single target.

**Fig. 6:**
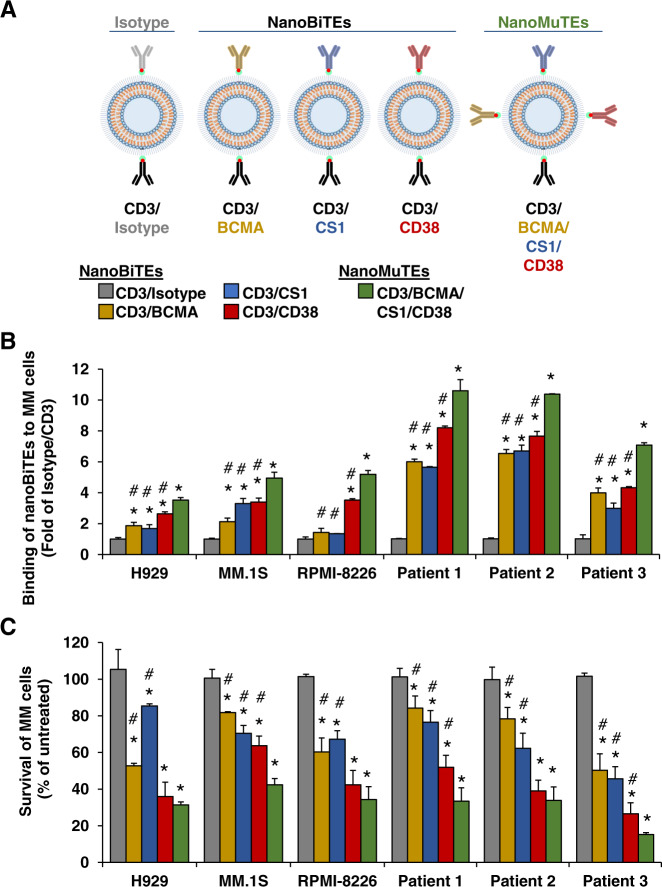
Development and biological function of nanoparticle multispecific T-cell engagers (nanoMuTEs). **A** Illustrations of the isotype/CD3, BCMA/CD3, CS1/CD3, CD38/CD3 nanoBiTEs, and BCMA/CS1/CD38/CD3 nanoMuTEs. **B** Binding of the nanoBiTEs and nanoMuTEs to MM cell lines and primary cells (*n* = 3; means ± SD). **C** The effect of nanoBiTEs and nanoMuTEs on the killing of MM cells by T cells (*n* = 4; means ± SD). One-way and two-way ANOVA was used; statistical significance (*p*  < 0.05) was indicated using two symbols (* and #); specifically, an asterisk symbol (*) represents significance between the nanoBiTE and isotype/CD3, and a hash symbol (#) represents significance between the nanoBiTE and nanoMuTE.

Therefore, we developed a nanoparticle that targets multiple cancer antigens simultaneously by conjugating multiple mAbs against multiple cancer antigens for T-cell engagement (nanoMuTEs; Fig. 6A). We hypothesized that nanoMuTEs will target multiple clones simultaneously, prevent antigen-less tumor escape, and be more efficacious than targeting individual antigens.

We tested the binding of three different BCMA/CD3, CS1/CD3, and CD38/CD3 nanoBiTEs (each targeting one antigen) and BCMA/CS1/CD38/CD3 nanoMuTEs (targeting all three antigens) to MM cells. Each nanoBiTE bound to MM cells more than the isotype/CD3 nanoBiTE, in correlation with the surface expression of each antigen; nanoMuTEs showed higher binding compared to each nanoBiTE alone (Fig. 6B).

We further tested T-cell-induced killing of MM cells by nanoBiTEs and nanoMuTEs in the 3DTEBM. Each nanoBiTEs induced more MM killing compared to isotype/CD3, while nanoMuTEs induced more MM killing compared to each nanoBiTE (Fig. 6C). We also tested T-cell activation. Activation of CD4 and CD8 T cells (Supplementary Fig. 3Ai, ii, respectively) was higher after treatment with each nanoBiTE compared to isotype/CD3, while activation after treatment with nanoMuTEs was higher than each nanoBiTE. CD8 T cells showed higher activation compared to CD4 T cells when treated with any of the nanoBiTEs or nanoMuTEs. In addition, we investigated the presence of cytokines following treatment with nanoBiTEs or nanoMuTEs (Supplementary Fig. 3Bi, ii). The presence of IL-2, IL-6, IL-10, TNF-α, and IFN-γ is significantly greater when treated with each nanoBiTE compared to isotype/CD3; nanoMuTEs induced greater secretion than the nanoBiTEs.

**Fig. 7:**
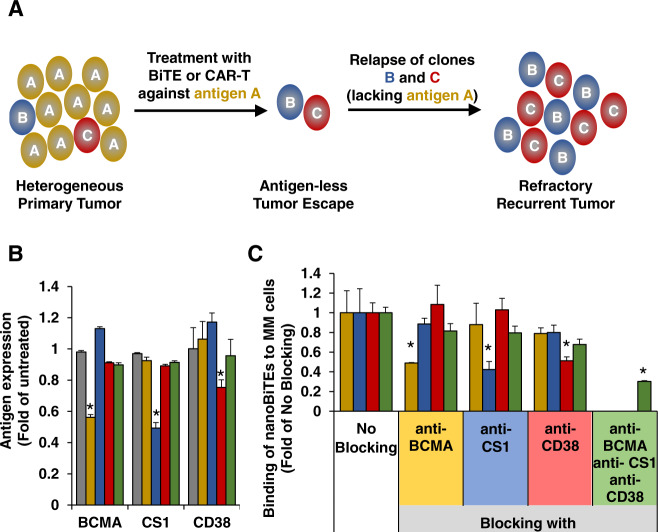
Circumventing antigen-less tumor escape with nanoMuTEs in vitro. **A** Schematic of the mechanism of tumor escape after treatment with immunotherapies targeting one antigen due to development of antigen-less tumor clones which cause relapse of the disease. **B** The effect of nanoBiTEs and nanoMuTEs on the expression of BCMA, CS1, and CD38 on MM cells remaining following treatment (*n* = 4; means ± SD). **C** The effect of blocking tumor antigens (BCMA, CS1, and CD38) on the binding of nanoBiTEs and nanoMuTEs to MM cells (*n* = 3; means ± SD). One-way and two-way ANOVA was used; statistical significance (*p*  < 0.05) was indicated using an asterisk symbol (*) which represents significance between the nanoBiTE and isotype/CD3 in panel B and represents significance between the nanoBiTE with no blocking and nanoBiTE with blocking in panel C.

Next, we developed antigen-less clones by testing the effect of nanoBiTEs and nanoMuTEs on the expression of antigens on MM cells (Fig. 7A). When treated with BCMA/CD3, CS1/CD3, or CD38/CD3, the expression of BCMA, CS1, and CD38 in the whole MM cell population was decreased, respectively (Fig. 7B), but not affected by the nanoBiTEs with other targets. The decrease can be attributed to killing of the population with high expression of the specific antigen or downregulation of the specific antigen on the cells, both of which contribute to the development of antigen-less populations. In contrast, the treatment with nanoMuTEs did not generate a population with lower expression of any of the three antigens, which suggests that treatment with nanoMuTEs will not cause antigen-less tumor escape and create a better therapeutic strategy.

We next investigated the effect of blocking (as a model for downregulation) of BCMA, CD38, and CS1 on the binding of nanoBiTEs and nanoMuTE to MM cells. The binding of each of the nanoBiTEs was reduced when the antigens on the cells were blocked with the respective blocking antibody against the antigen that it is targeting. In contrast, no significant decrease of the binding of nanoMuTEs was observed when treated with any of the antibodies blocking alone, likely because the binding was facilitated through other antigens. Binding of nanoMuTEs was decreased when treated with a combination of the three blocking antibodies (Fig. 7C). This demonstrates that downregulation (or loss) of an antigen will reduce the binding (and hence the efficacy) of the nanoBiTE, as observed clinically with the treatment with CAR-T cells and BiTEs, but did not affect the binding of nanoMuTEs, which creates a better therapeutic strategy.

**Fig. 8:**
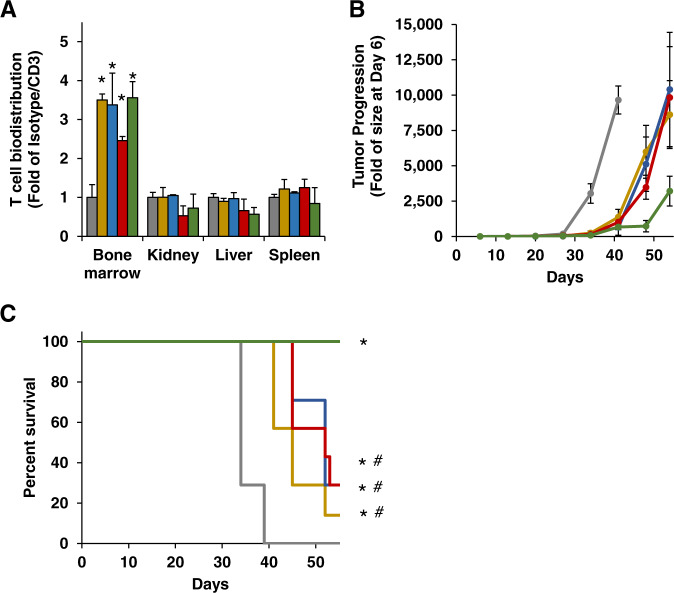
Circumventing antigen-less tumor escape with nanoMuTEs in vivo. **A** Biodistribution of T cells following 24 h in vivo (*n* = 3; means ± SD). **B** The effect of nanoBiTEs and nanoMuTEs on the progression of MM tumors in vivo (*n* = 7; means ± SEM). **C** The effect of nanoBiTEs and nanoMuTEs on the survival of MM-bearing mice (*n* = 7). One-way and two-way ANOVA was used to assess **A**. Log-rank test was used to compare the Kaplan–Meier curves; statistical significance (*p*  < 0.05) was indicated using two symbols (* and #); specifically, an asterisk symbol (*) represents significance between the nanoBiTE and isotype/CD3, and a hash symbol (#) represents significance between the nanoBiTE and nanoMuTE.

We also investigated the biodistribution of nanoBiTEs/nanoMuTEs and T cells following 24 h (Supplementary Fig. 4 and Fig. 8A, respectively). We see specific accumulation of nanoBiTEs and nanoMuTEs at the tumor site (BM) compared to isotype/CD3. Consequently, T cells were specifically engaged to the tumor site following treatment with nanoBiTEs or nanoMuTEs compared to isotype/CD3. Next, the pharmacokinetic profile of each of the nanoBiTEs and nanoMuTEs was similar to the CD20/CD3 nanoBiTEs with a half-life of ~50–60 h (Supplementary Fig. 5). To demonstrate the therapeutic efficacy of the nanoBiTEs and nanoMuTEs in vivo, we used an aggressive xenograft MM model by injecting MM.1S IV (with humanized T cells), which kills the mice in less than 4–5 weeks after injection, if not treated, and represents the clinically aggressive/relapsed form of the disease. Treatment with isotype/CD3 nanoBiTEs showed fast tumor progression and death of the cohort within 40 days (Fig. 8B and Supplementary Fig. 6). Treatment of each of the nanoBiTEs targeting one antigen (BCMA, CS1, or CD38) resulted in delayed tumor progression and prolonged survival, while the treatment with the nanoMuTEs induced longer tumor progression delay and resulted in survival of the entire cohort till 55 days (Fig. 8C).

Our study successfully shows the proof-of-concept of redirecting T cells to cancer using nanoparticles. The nanoBiTEs/nanoMuTEs used for WM and MM were able to induce T-cell-mediated cancer cell killing. The effect of the CD20/CD3 nanoBiTEs for the treatment of WM was significantly more profound than the nanoBiTEs/nanoMuTEs used for MM; CD20/CD3 cured the WM xenograft murine model, whereas the nanoBiTEs/nanoMuTEs prolonged survival of MM mice by only 10–20 days. This is, likely, due to the difference in antigen level and presence on each cancer type; expression of CD20 was prevalent in the vast majority of WM cells, while the expression of BCMA, CS1, and CD38 was variable on MM. Moreover, the intensity of the expression of CD20 on WM cells was 2–3 orders of magnitude higher than BCMA, CS1, and CD38 on MM.

In conclusion, the nanoBiTE/nanoMuTE platform uses nanotechnology to provide a relatively easy-to-make and off-the-shelf solution to circumvent the major limitations of the current immunotherapy technologies (CAR-T cells and BiTEs). It takes advantage of the established high specificity of mAbs to better navigate the robust immune response to eliminate cancer. In this instance, it would be easy to modify this system to generate a new nanoBITE as an immunotherapy to target any cancer type by using existing or new mAbs that have the ability to specifically bind to the cancer cells of interest. The flexibility of the nanoparticle-based immuno-engaging technology provides a general platform with groundbreaking translational potential for developing easy-to-make, specific, and efficacious immunotherapy for cancer in general.

## Methods

### Materials and reagents

All biotinylated and fluorescent antibodies, human CD138 microbeads, and Pan T Cell Isolation Kits were purchased from Miltenyi Biotec (Bergisch Gladbach, Germany). DMEM, RPMI-1640, L-glutamine, penicillin–streptomycin, and phosphate buffered saline (PBS) were purchased from Corning (Corning, NY). Fetal bovine serum, live-cell dyes, lipophilic tracers, collagenase, and counting beads were purchased from Life Technologies (Carlsbad, CA). 1,2-dipalmitoyl-sn- glycero-3-phosphocholine (DPPC), 1,2-distearoyl-sn-glycero-3-phosphoethanolamine-N- [amino(polyethylene glycol)-2000] (DSPE-PEG2000), and polycarbonate membranes were purchased from Avanti Polar Lipids (Alabaster, AL). Cholesterol and chloroform were purchased from Millipore Sigma (Burlington, MA). Streptavidin conjugation kit was purchased from Abcam (Cambridge, UK). Human Cytokine Array Q1 was purchased from RayBiotech (Peachtree Corners, GA). All mice used in this study were NCG (strain: 572), female, 50–56 days old, and purchased from Charles River (Wilmington, MA). All mice experiments in this study were in compliance with the Institutional Animal Care and Use Committee at Washington University.

### Cells

H929, MM.1S, and RPMI-8226 were purchased and authenticated by American Type Culture Collection (Manassas, Virginia). All cell lines were tested for mycoplasma contamination. BCWM.1 and MWCL.1 were a gift from Irene Ghobrial. Primary BM samples were isolated from MM patients at Washington University School of Medicine (IRB # 201102270) and subsequently selected for MM cells with the use of CD138 human microbeads. Informed consent was obtained from all individuals in accordance with the Declaration of Helsinki. Normal donor peripheral blood mononuclear cells (PBMCs) were isolated from healthy donors using Ficoll centrifugation [43] and subsequently separated for T cells using a human Pan T Cell Isolation Kit (Miltenyi Biotec). Hs505.T cells were cultured in DMEM with 4.5 g/l glucose and L-glutamine with the addition of addition of 10% fetal bovine serum and 1% penicillin–streptomycin. The other cell lines were cultured in RPMI-1640 with the addition of 10% fetal bovine serum, 2 mM of L-glutamine, and 1% penicillin–streptomycin.

### Preparation and characterization of the nanoBiTEs and nanoMuTEs

As described in Fig. 1B, nanoBiTEs consisted of three components: cholesterol, DPPC, and DSPE-PEG2000 with a mass ratio equivalent to 30:65:5, respectively. Lipids were mixed and solubilized in chloroform, and evaporated to form a thin film [44, 45]. Then, the film was hydrated with PBS, and the resulting suspension was extruded using the Avestin LiposoFast LF-50 (Ottawa, ON, Canada) with 100 nm polycarbonate membranes to yield unilamellar liposomes. Streptavidin was conjugated to the amine groups on the surface of the liposomes according to the protocol of the manufacturer (Abcam), to activate the liposomes. Biotinylated antibodies were added to bind to the streptavidin for targeting, as previously described [46]. For detailed amounts of each reagent used, please see Supplementary Table 1. Malvern Zetasizer Nano ZS90 (Malvern, Worcestershire, UK) was used to determine zeta-potential, diameter, and polydispersity index of the each preparation (see Supplementary Table 2 for details).

### Pharmacokinetics of nanoBiTEs and nanoMuTEs

Each nanoBiTE or nanoMuTE was stained with a fluorescent tracer (DiD and injected IV injection to NSG mice at .5 mg/mouse (*n* = 3 for each formulation). Blood (50 μl) was taken from the tail vein of each mouse before treatment, and 0.25, 6, 24, 48, 72, and 96 h after treatment. Fluorescence of whole blood or plasma was measured at 644/665 nm using a SpectraMax i3 plate reader (Molecular Devices, San Jose, CA). Half-life was calculated using polynomial regression.

### Cell surface protein expression analysis

Cell lines or primary CD138+ MM cells were incubated with APC-anti-CD20, APC-anti-BCMA, APC-anti-CS1, or APC-anti-CD38 antibodies in 4 °C for 1 h; then washed, spun down, resuspended in 100 μl and analyzed by flow cytometry using MACSQuant Analyzer 10 with Ex = 635 nm and Em = 655–730 nm [47]. Cells were gated using FSC and SSC, and analyzed for relative mean fluorescent intensity (RMFI) of APC using BD FlowJo Software [48].

### Liposome binding and binding following antigen loss in vitro

Each nanoBiTE or nanoMuTE was stained with a fluorescent tracer DiO. Cell lines and primary cells (30,000 cells in 100 μl for each data point) were treated with or without isotype/CD3, nanoBiTEs, or nanoMuTEs (3.7 nM) for 2 h at 37 °C. In some cases (for mimicking antigen downregulation), cells were treated with 33.3 nM of anti-BCMA, CS1, and/or CD38 antibody of the same clone for 1 h prior to the 2-h treatment with nanoBiTEs or nanoMuTEs. Following the 2-h treatment with nanoBiTEs or nanoMuTEs, the cells were stained with anti-BCMA, CS1, or CD38 of a different clone for 1 h. Then, cells were spun down, washed with PBS, resuspended in 100 μl and analyzed by flow cytometry using MACSQuant Analyzer 10 with Ex = 488 nm and Em = 525/50 nm. Cells were gated using forward and side scatter, and analyzed for MFI of DiO using BD FlowJo Software.

### 3DTEBM culture system

The culture’s cellular content can be customized by inclusion of various cell populations. For testing patient samples, BM mononuclear cells were used as a whole, including the primary cancer cells and T cells. 3DTEBM was established by cross-linking fibrinogen in patient BM supernatant using CaCl_2_, as previously described [28]. Briefly, for testing cell lines, 30,000 cancer cells were combined with 30,000 T cells; for primary cells 100,000 BM mononuclear cells were used as whole. Cells were suspended in BM supernatant which was then cross-linked with CaCl_2_ to form the 3D matrix. The 3DTEBM was supplemented with media on top and incubated at 37 °C for 4 days. At time of analysis, the scaffolds were digested with collagenase (Gibco, Life Technologies) for 2 h at 37 °C; cells were retrieved, washed, and subjected to flow cytometry analysis.

For the development of antigen-less populations, the above procedure was followed and the remaining cells were incubated with APC-anti-BCMA, APC-anti-CS1, or APC-anti-CD38 antibodies in 4 °C for 1 h; then washed, spun down, resuspended in 100 μl and analyzed by flow cytometry using MACSQuant Analyzer 10 with Ex = 635 nm and Em = 655–730 nm. Cells were gated using FSC and SSC, and analyzed for MFI of APC using BD FlowJo Software.

### Cell survival

Cell lines (prelabeled with fluorescent tracer DiO) and primary cells were incubated with T cells in 3DTEBM and treated with or without isotype/CD3, nanoBiTEs, or nanoMuTEs at a concentration of 3.7 nM for 4 days. Before digestion of the matrix, 5 μl of counting beads (Miltenyi Biotec) were added to the culture. The matrix was then digested, cells were retrieved, and analyzed by flow cytometry using MACSQuant Analyzer 10. For cell lines, the number of tumor cells analyzed as DiO+ cells and normalized to the number of counting beads using BD FlowJo Software. For primary cells, MM cells were identified as CD38+/CD3−/CD14−/CD16−/CD19−/CD123−, as previously described [49], and the number of MM primary cells was normalized to the number of counting beads using BD FlowJo Software. For the analysis of WM killing without T cells, the above procedure was mimicked except without including T cells in 3DTEBM.

### Activation of T cells

Cells were in 3DTEBM and treated with or without isotype/CD3, nanoBiTEs, or nanoMuTEs at a concentration of 3.7 nM for 4 days. Then, cultures were digested, and the cells were retrieved and incubated with PE anti-CD3, FITC anti-CD4, Violet anti-CD8, and APC-anti-CD69 antibodies for 1 h in 4 °C, washed with PBS, spun down, and suspended in PBS again. These samples were analyzed by flow cytometer using MACSQuant Analyzer 10 with Ex = 488, 488, 405, and 635 nm and Em = 585/40, 525/50, 450/50, 655–730 nm, respectively. Cells were gated using FSC and SSC followed by double positive CD3+/CD4+ or CD3+/CD8+, both of which were analyzed for % of cells positive for CD69 using BD FlowJo Software.

For cytokine secretion, the supernatant was kept and the 3DTEBM was digested for 2 h using collagenase following the 4-day incubation period. Once the 3DTEBM was digested and samples were spun down, the supernatant (with collagenase) was then added to the supernatant collected earlier. Subsequently, the samples were analyzed for cytokine presence following the manufacturer’s protocol and scanned using the InnoScan 710 microarray fluorescence scanner (Innopsys) by the manufacturer of the cytokine array.

### NanoBiTE/nanoMuTE and T-cell biodistribution, tumor efficacy, and survival in vivo

For all animal studies, mice were randomized into groups and no blinding was done in this study. For biodistribution, human MM.1S-CBR cells (2 × 10^6^/mouse) were injected IV to NSG mice to generate the MM tumor models. PBMCs were isolated from healthy human donors using Ficoll centrifugation and subsequently separated for T cells using a human Pan T Cell Isolation Kit (Miltenyi Biotec), as previously described [43]. T cells (5 × 10^6^/mouse) were stained with calcein violet and injected IV to each mouse 3 weeks following propagation of the MM cells. One hour post T-cell injection, mice were treated IV with isotype/CD3, nanoBiTEs, or nanoMuTEs stained with DiD (0.5 mg/mouse). Organs were extracted 24 h later and analyzed via flow cytometry.

For tumor efficacy and survival, human BCWM.1 or MM.1S luciferase cells (2 × 10^6^/mouse) were injected IV to NSG mice to generate the WM or MM tumor models, respectively [50]. T cells (5 × 10^6^/mouse) were injected IV to each mouse 7 days after the injection of tumor cells. One hour post T-cell injection, mice were treated IV with isotype/CD3, nanoBiTEs, or nanoMuTEs (0.5 mg/mouse) and weekly thereafter.

For tumor progression, mice were imaged weekly using bioluminescent imaging. Mice were injected with D-luciferin (150 μg/kg) intraperitoneally, and tumor burden was detected using an IVIS 50 bioluminescence imaging system (PerkinElmer, Waltham, MA) 10 min post luciferin injection, and images were analyzed using Living Image 2.6 software (PerkinElmer). For survival, mice were monitored on a daily basis to record survival.

### Gene expression analysis

Gene expression data on MM patients were extracted from the previously published literature [51] describing data from 600 newly diagnosed MM patients, in which plasma cells were subsequently selected using anti-CD138 beads and mRNA gene expression was performed using the Affymetrix U133 Plus 2.0 microarray platform (Santa Clara, CA) and analyzed using the Affymetrix Microarray Suite GCOS1.1. BCMA, CS1, and CD38 gene expression was analyzed and plotted using Python.

### Statistical analyses

All in vitro experiments in this study was independently replicated three times. Sample size for laboratory animals was estimated using published guidelines [52]. In vitro experiments were performed in quadruplicates, and in vivo experiments consisted of seven mice each; data from in vitro and in vivo experiments were expressed as means ± standard deviation. Data normality was analyzed using residuals, and variance similarity across groups was also analyzed by examining the expected variance of each group. Statistical significance was analyzed using a Student’s *t* test and one-way or two-way analysis of variance (ANOVA). Log-rank test was used to compare the Kaplan–Meier curves. P values less than 0.05 were used to indicate statistically significant differences.

## Supplementary information

Supplementary Table 1

Supplementary Table 2

Supplementary Figure 1

Supplementary Figure 2

Supplementary Figure 3

Supplementary Figure 4

Supplementary Figure 5

Supplementary Figure 6

## Data Availability

All data generated are available upon request.
