# Supplementary material for: Nanoparticle T-cell engagers as a modular platform for cancer immunotherapy
Source: Leukemia. 2021 Jan 21;35(8):2346–57. doi: 10.1038/s41375-021-01127-2 (PMC8292428; doi:10.1038/s41375-021-01127-2)
Supplement: Supplementary file 1 — Supplementary Table 1 [file 41375_2021_1127_MOESM1_ESM.pdf]

Supplementary Table 1

	Lipids				Streptavidin	Biotinylated Antibodies					
	DPPC	Cholesterol	DSPE-PEG-NH2	18:0 PC-Et-NH2		CD3	Isotype	CD20	BCMA	CS1	CD38
Cat #	850355	C8667	880128	860376	ab102921	130-113-137	130-113-448	130-111-336	130-104-500	130-099-575	130-113-430
Concentration (mg/mL)						0.022	0.020	0.150	0.110	0.044	0.022
Unit	mg	mg	mg	mg	mg	µL	µL	µL	µL	µL	µL
Pegylated Liposomes	13	3.2	3.8		0.1						
CD3 Liposomes (1 ab/liposome)	13	3.2	3.8		0.1	874					
CD3 Liposomes (3 ab/liposome)	13	3.2	3.8		0.1	2622					
CD3 Liposomes (10 ab/liposome)	13	3.2	3.8		0.1	8740					
CD20 Liposomes (1 ab/liposome)	13	3.2	3.8		0.1			146			
CD20 Liposomes (3 ab/liposome)	13	3.2	3.8		0.1			438			
CD20 Liposomes (10 ab/liposome)	13	3.2	3.8		0.1			1460			
BCMA Liposomes (1 ab/liposome)	13	3.2	3.8		0.1				199		
BCMA Liposomes (3 ab/liposome)	13	3.2	3.8		0.1				597		
BCMA Liposomes (10 ab/liposome)	13	3.2	3.8		0.1				1990		
CS1 Liposomes (1 ab/liposome)	13	3.2	3.8		0.1					497	
CS1 Liposomes (3 ab/liposome)	13	3.2	3.8		0.1					1491	
CS1 Liposomes (10 ab/liposome)	13	3.2	3.8		0.1					4970	
CD38 Liposomes (1 ab/liposome)	13	3.2	3.8		0.1						994
CD38 Liposomes (3 ab/liposome)	13	3.2	3.8		0.1						2982
CD38 Liposomes (10 ab/liposome)	13	3.2	3.8		0.1						9940
Isotype/CD3 nanoBiTEs	13	3.2	3.8		0.1	874	1092				
CD20/CD3 nanoBiTEs	13	3.2	3.8		0.1	874		146			
BCMA/CD3 nanoBiTEs	13	3.2	3.8		0.1	874			199		
CS1/CD3 nanoBiTEs	13	3.2	3.8		0.1	874				497	
CD38/CD3 nanoBiTEs	13	3.2	3.8		0.1	874					994
BCMA/CS1/CD38/CD3 nanoMuTEs	13	3.2	3.8		0.1	874			199	497	994
CD20/CD3 nanoBiTEs (no PEG)	13	3.2		1.1	0.1	874		146			

Supplementary Table 1. Amounts of lipids and antibodies used to make each specific liposome formulation.
