# Supplementary material for: Nanoparticle T-cell engagers as a modular platform for cancer immunotherapy
Source: Leukemia. 2021 Jan 21;35(8):2346–57. doi: 10.1038/s41375-021-01127-2 (PMC8292428; doi:10.1038/s41375-021-01127-2)
Supplement: Supplementary file 2 — Supplementary Table 2 [file 41375_2021_1127_MOESM2_ESM.pdf]

Supplementary Table 2

Formulation	Mean Size (nm)	Polydispersity Index	Zeta Potential (mV)
Isotype/CD3	127.9 ± 3.6	0.066 ± 0.030	0.67 ± 0.32
CD20/CD3	128.1 ± 11.3	0.085 ± 0.034	1.41 ± 0.70
BCMA/CD3	133.2 ± 9.3	0.091 ± 0.017	-0.02 ± 0.05
CS1/CD3	123.4 ± 8.4	0.082 ± 0.040	0.61 ± 0.50
CD38/CD3	125.4 ± 8.3	0.107 ± 0.006	0.39 ± 0.38
BCMA/CS1/CD38/CD3	125.2 ± 0.5	0.047 ± 0.021	0.84 ± 0.34

Mean ± standard deviation

Supplementary Table 2. Parameters (size, polydispersity index, and zeta potential) for each nanoBiTE or nanoMuTE.
