# Supplementary material for: Nanoparticle T-cell engagers as a modular platform for cancer immunotherapy
Source: Leukemia. 2021 Jan 21;35(8):2346–57. doi: 10.1038/s41375-021-01127-2 (PMC8292428; doi:10.1038/s41375-021-01127-2)
Supplement: Supplementary file 3 — Supplementary Figure 1 [file 41375_2021_1127_MOESM3_ESM.pdf]

# Supplementary Figure 1

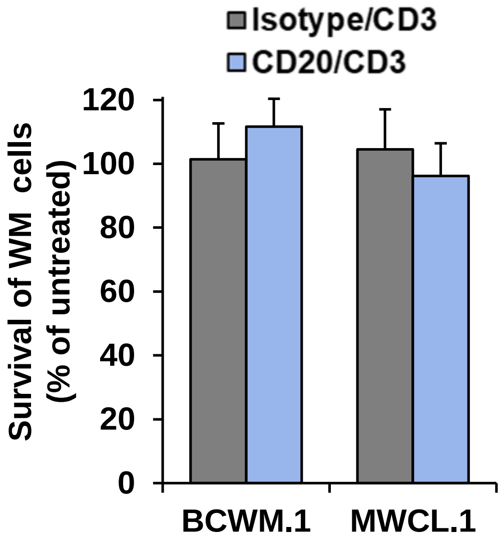


**Supplementary Fig. 1. The effect of Isotype/CD3 and CD20/CD3 nanoBiTEs on the killing of WM cells without T cells (n=3; means ± SD).**
