# Supplementary material for: Nanoparticle T-cell engagers as a modular platform for cancer immunotherapy
Source: Leukemia. 2021 Jan 21;35(8):2346–57. doi: 10.1038/s41375-021-01127-2 (PMC8292428; doi:10.1038/s41375-021-01127-2)
Supplement: Supplementary file 4 — Supplementary Figure 2 [file 41375_2021_1127_MOESM4_ESM.pdf]

Supplementary Figure 2

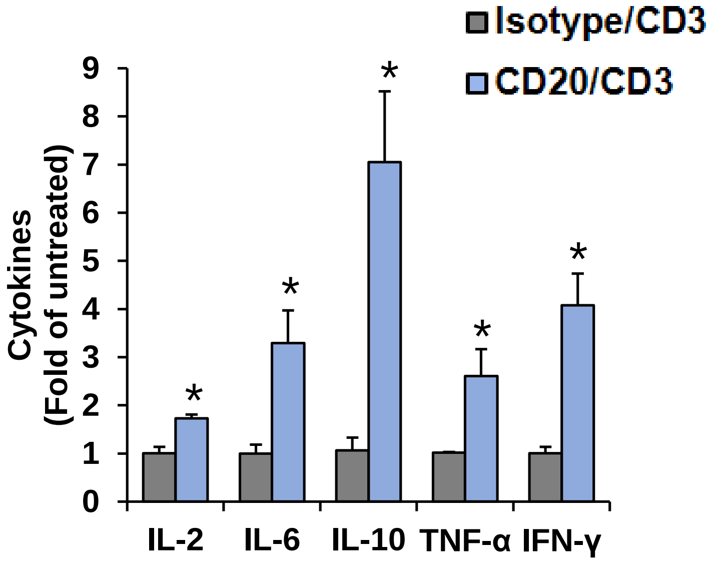

Supplementary Fig. 2. Cytokine secretions following treatment of WM cells with Isotype/CD3 and CD20/CD3 (n=5; means ± SD).
