# Supplementary material for: Nanoparticle T-cell engagers as a modular platform for cancer immunotherapy
Source: Leukemia. 2021 Jan 21;35(8):2346–57. doi: 10.1038/s41375-021-01127-2 (PMC8292428; doi:10.1038/s41375-021-01127-2)
Supplement: Supplementary file 5 — Supplementary Figure 3 [file 41375_2021_1127_MOESM5_ESM.pdf]

Supplementary Figure 3

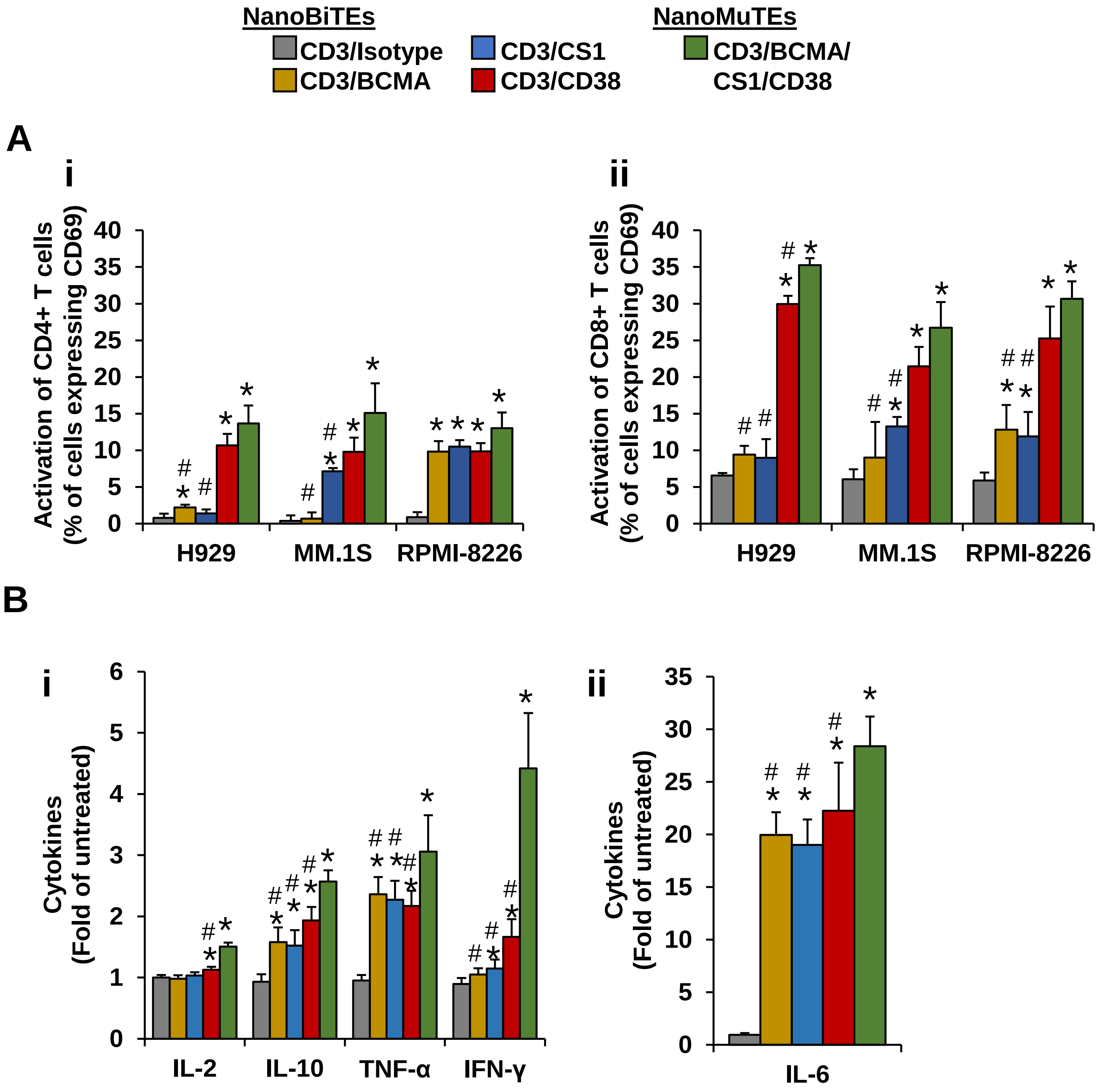

Supplementary Fig. 3. Ai. and ii. The effect of nanoBiTEs and nanoMuTEs on the expression of CD69 on CD4+ and CD8+ T cells, respectively (n=4; means ± SD). B. i. and ii. Cytokine secretions following treatment of MM with nanoBiTEs or nanoMuTEs (n=5; means ± SD).
