# Supplementary material for: Nanoparticle T-cell engagers as a modular platform for cancer immunotherapy
Source: Leukemia. 2021 Jan 21;35(8):2346–57. doi: 10.1038/s41375-021-01127-2 (PMC8292428; doi:10.1038/s41375-021-01127-2)
Supplement: Supplementary file 8 — Supplementary Figure 6 [file 41375_2021_1127_MOESM8_ESM.pdf]

Supplementary Figure 6

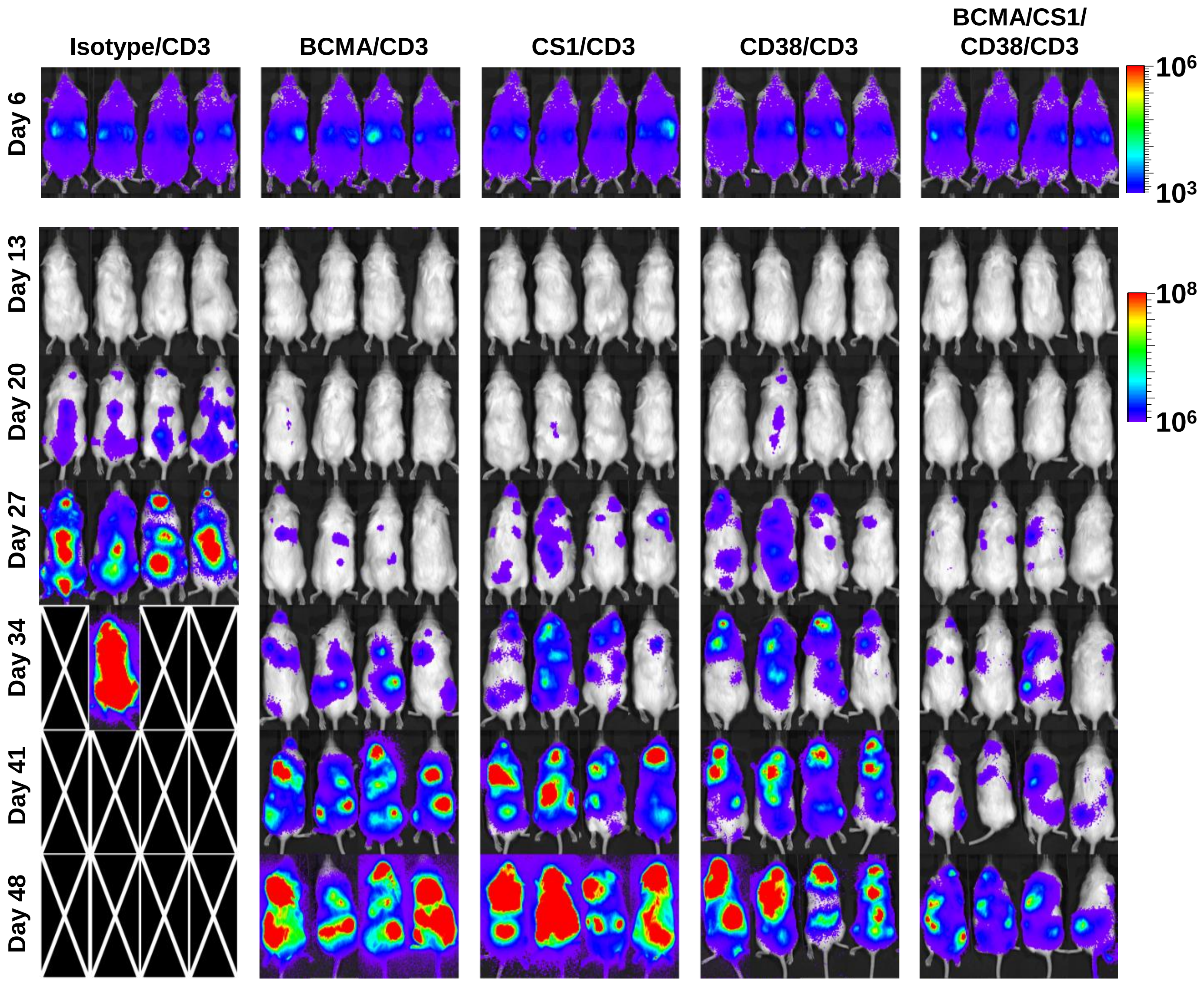

Supplementary Fig. 6. The effect of nanoBiTEs and nanoMuTEs on the progression of MM tumors in vivo (n=7).
